# Visualizing statistical significance of disease clusters using cartograms

**DOI:** 10.1186/s12942-017-0093-9

**Published:** 2017-05-15

**Authors:** Barry J. Kronenfeld, David W. S. Wong

**Affiliations:** 1 Department of Geology and Geography, Eastern Illinois University, 600 Lincoln Avenue, Charleston, IL 61920-3099 USA; 2 Geography and Geoinformation Science, George Mason University, 4400 University Drive, Fairfax, VA 22030 USA

**Keywords:** Cartograms, Density equalizing maps, Disease mapping, Scan statistics, Geovisual analytics

## Abstract

**Background:**

Health officials and epidemiological researchers often use maps of disease rates to identify potential disease clusters. Because these maps exaggerate the prominence of low-density districts and hide potential clusters in urban (high-density) areas, many researchers have used density-equalizing maps (cartograms) as a basis for epidemiological mapping. However, we do not have existing guidelines for visual assessment of statistical uncertainty. To address this shortcoming, we develop techniques for visual determination of statistical significance of clusters spanning one or more districts on a cartogram. We developed the techniques within a geovisual analytics framework that does not rely on automated significance testing, and can therefore facilitate visual analysis to detect clusters that automated techniques might miss.

**Results:**

On a cartogram of the at-risk population, the statistical significance of a disease cluster is determinate from the rate, area and shape of the cluster under standard hypothesis testing scenarios. We develop formulae to determine, for a given rate, the area required for statistical significance of a priori and a posteriori designated regions under certain test assumptions. Uniquely, our approach enables dynamic inference of aggregate regions formed by combining individual districts. The method is implemented in interactive tools that provide choropleth mapping, automated legend construction and dynamic search tools to facilitate cluster detection and assessment of the validity of tested assumptions. A case study of leukemia incidence analysis in California demonstrates the ability to visually distinguish between statistically significant and insignificant regions.

**Conclusion:**

The proposed geovisual analytics approach enables intuitive visual assessment of statistical significance of arbitrarily defined regions on a cartogram. Our research prompts a broader discussion of the role of geovisual exploratory analyses in disease mapping and the appropriate framework for visually assessing the statistical significance of spatial clusters.

**Electronic supplementary material:**

The online version of this article (doi:10.1186/s12942-017-0093-9) contains supplementary material, which is available to authorized users.

## Background

In epidemiological analysis, there is a long history of mapping disease and related health and socio-demographic data. Maps are used to explore spatial patterns and identify potential causal factors, which then may warrant further investigation by cohort and case–control studies [1]. Because human visual perception is adept at spatial pattern recognition, epidemiological maps may facilitate detection of clusters that might be missed using confirmatory statistical techniques in which potential cluster regions must be designated a priori. Maps also provide spatial context and present data in a visual form that is accessible to the public. The need to respond to public concern has spurred development of automated systems to construct maps of disease occurrence [2, 3].

Though maps can help identify disease clusters, there is much controversy over their use. Map readers likely see clusters that may just be the results of random variation, or a mild degree of clustering of similar values. Yet further investigation to determine their statistical significance requires substantial resources [4, 5]. For this reason, the communication of statistical uncertainty is an important problem in disease mapping [1, 2, 6].

Although the problem of visualizing uncertainty is broadly recognized by cartographers [7], it is particularly significant in disease mapping due to the rareness of disease events and the non-uniform spatial distribution of the underlying population. These factors compound each other, as a standard map may place disproportionate emphasis on geographically large but sparsely populated districts, and these same districts will tend to have relatively unreliable observed disease rates as reflected by their large standard errors or related statistics [1]. Map readers will therefore have difficulty visually distinguishing between statistically significant clusters and areas that have high disease rates but are not statistically significant due to small sample size.

One method to avoid placing disproportionate visual emphasis on geographically large but sparsely populated districts is to use a density-equalizing map, or “cartogram”. A cartogram is a map in which district shapes are altered such that their size is proportional to a designated variable, typically population. This results in a uniform density of the population variable, and so cartograms have also been referred to as density-equalizing maps [8]. When used as a basis for disease mapping, population cartograms reduce the size, and therefore the visual prominence, of low-population districts in which high disease rates might occur due to chance alone [9–12].

While visual emphasis can influence human perception, a more concrete approach is needed to communicate statistical uncertainty. In this paper, we extend the cartogram visualization framework to develop a formal visual assessment method for determining the statistical significance of observed disease rates over user-defined regions. Our approach is based on the fact that statistical variance is normally an inverse function of population, and therefore statistical significance is determinate from the population and observed disease rate of a district. Given this, we propose using a cartogram to show population combined with standard choropleth symbolization to show observed disease rates, thereby visually communicating the necessary data to determine statistical significance. The resulting visual framework does not require use of coincident or overlapping symbols that have been shown to reduce map readers’ ability to effectively perform analysis tasks.

An advantage of the proposed geovisual analytics framework is that it supports on-the-fly determination of statistical significance for user-defined regions consisting of multiple districts or portions of districts. As such, it supports truly exploratory analysis, exploiting the map reader’s visual perception and spatial cognition to identify and assess clusters that are unknown in advance and might not be detected by automated search techniques. Most standard tests of statistical significance apply to a priori defined districts and are therefore problematic in an exploratory framework due to the issue of multiple hypothesis testing. To support estimation of the statistical significance of a posteriori defined districts, we use scan statistics that provide a stricter test based on the likelihood of a given cluster occurring anywhere on the map under the null hypothesis. A measure of compactness is further used to mitigate problems introduced when irregularly-shaped clusters are considered.

### Statistical significance in maps

The importance of communicating uncertainty and/or statistical significance associated with disease rates is widely appreciated in the epidemiological community. While any location with a high rate represents a potential area of concern, areas in which the observed rate is unlikely the result of random variation should be given higher priority than those that fall within statistical confidence intervals [6, 13]. Despite this recognition, constructing maps that effectively communicate observed rates and associated uncertainty simultaneously has proven to be a difficult challenge [7]. Attempts to meet this challenge have included both statistical and visual approaches, each of which has strengths and weaknesses.

Statistical approaches involve the construction of a single variable that communicates some degree of both the estimated magnitude of risk (i.e. effect size) and associated uncertainty (i.e. significance). A simple approach is to map statistical significance values rather than crude disease rates. The drawback of this approach is that the influences of disease rate and sample size cannot be distinguished [14]. For example, the same significance value might indicate a very high disease rate in a small sample or a slightly elevated disease rate in a large sample. An alternative is to use Bayesian techniques to adjust the relative risk estimates on the basis of sample size and/or neighborhood [14, 15]. In this approach, a prior risk ratio estimate is constructed based on either the overall rate or the rate observed in a predefined neighborhood around each district. This prior value is then updated based on the observed rate and sample size in each district, again sometimes including neighboring districts, resulting in a posterior risk estimate that is moderated by sample size and neighborhood effects. Bayesian estimation techniques are now widely used in health mapping [2], but map readers must be aware that the smoothing effect of these techniques reduces effect size in favor of stable estimates [1]. Specifically, the rate estimate shown in each district will be a weighted average of the actual rate in that district, rates in nearby districts and the overall mean rate [14]. The exact nature of this tradeoff is dependent on the underlying model [15].

In contrast to the statistical approach that combines information from the observed rate and sample size into a single significance value, the visual approach attempts to communicate two values (e.g. rates and significance levels) simultaneously. Since using two maps to display the two sets of values requires additional space or a scale reduction of maps, a preferable method is to put both sets of values on a single map using symbol overlay. Several health agencies produce online maps with texture overlays (e.g. stipple marks, diagonal or cross-hatch lines) on top of a choropleth (sequential color-coded) map. There is, however, significant variation in the type of symbol used and their meaning. For example, the United Kingdom Small Area Health Statistics Unit’s Rapid Inquiry Facility (RIF) and the California Cancer Registry (CCR) both use prominent black dot stipple overlay, but in the RIF the overlay varies in density and indicates districts with rates that are more statistically significant [6], whereas the CCR uses a single stipple density to indicate districts with less stable estimates [3].

Cartographic studies are mixed in their assessment of the effectiveness of maps with overlapping symbols. Some studies have found that users perform worse on analysis tasks using a single map with uncertainty information overlaid onto each district than when uncertainty is presented separately on an adjacent map [7]. In addition, the approach is limited practically by the difficulty of placing texture symbols in small polygons/areas. However, other studies indicate that overlaid maps are more effective than adjacent maps to display both the mapped values and associated uncertainty statistics [16, 17]. An alternative is to take advantage of the multi-dimensional nature of color by using desaturation, transparency or blurring techniques to adjust an underlying sequential (e.g. light-to-dark) color scheme [7]. Some studies have found transparency to be effective at communicating uncertainty in a qualitative sense [18, 19], but the degree of precision with which users can decode transparency into a numerical value are still poorly understood.

A related problem is the disproportionate visual emphasis of districts based on their geographical area. Statistical districts that are relatively large in area may be sparsely populated, and can dominate visual perception despite representing only a small percentage of the population. In the USA, for example, 75% of the population resides in census tracts that occupy only 3.5% of the land area (according to 2010 data from the U.S. Census Bureau). This highly uneven distribution exacerbates the problem of communicating uncertainty, since random effects will be greater in the large but sparsely populated districts that tend to dominate visual perception [1]. In a map with many such districts, one or more district will inevitably appear as a disease hot-spot even when the distribution of disease occurrence is random across the population [6]. Conversely, heavily populated districts may not be visually prominent if they have small areas, even if their disease rates are high and statistically significant.

Another limitation of current methods is that they only encode uncertainty values at the level of the individual district. In practice, disease clusters may encompass multiple districts. However, it is impossible to visually determine the uncertainty or statistical significance associated with an aggregate region from symbols that encode values for each district individually.

## Methods

Many researchers have proposed cartograms as a solution to the problem of variable population density in health mapping [20, 21]. There have been two distinct approaches to cartogram usage in health research. In one approach, disease-related events are used as the designated cartogram variable, creating an *event cartogram*. On an event cartogram, districts with high numbers of events appear larger on the map. Recent studies have used event cartograms to visualize global incidence of drowning [22], mortality from ischaemic heart disease [23], as well as global variations in research effort related to health issues, including air pollution [24], traffic accidents [25], heat-related illness [26], yellow fever [27], influenza [28] and silicosis [29]. The visual effect of resizing countries on a world map based on the number of event occurrences is striking: countries with more events appear enlarged, while countries with fewer events are visually shrunken.

Unfortunately, many problems emerge with event cartograms when one realizes that map readers must disentangle the effects of land area, population, event frequencies and event rates. Any district with a high frequency of events relative to its land area will be enlarged on an event cartogram. This will include not only districts with high event rates per population (i.e. event hot-spots), but also districts with high population per unit land area (i.e. population hot-spots). Since usually we are interested in detecting event hot-spots, only the former cases are of interest. A variation on this approach is to use event rates instead of event counts as the cartogram variable [22, 28, 29]. However, using rates discards population size information and can therefore over-emphasize small population districts with high event rates that may be the result of random variation. The fact that small countries do not appear dramatically enlarged on published event rate cartograms is almost certainly due to deficiencies in cartogram algorithms that enlarge small areas sufficiently on the map [30].

The second approach differs from the first in that the underlying at-risk population, as opposed to the number of events, is designated as the cartogram variable, creating an *at*-*risk population cartogram*. Event locations or rates are then displayed on the top of the cartogram using standard mapping techniques, such as dot-density mapping or sequential choropleth color-coding. In this manner, the cartogram serves as a base map depicting the domain within which health events occur, rather than as a measure of the health events themselves. For example, in one study sheep scrapie occurrence and sampling rates were presented in dot maps and choropleth maps on a cartogram of the sheep population by county in Great Britain [9]. Although the focus is on scrapie incidence and detection, the cartogram allows map readers to quickly discern which counties have more sheep at risk. At-risk population cartograms have also been used to help contextualize rates of obesity in Canada [10], HIV incidence [11] and overall mortality [31] in Japan, and lung cancer cases in New York State [8].

Building on the same logic, at-risk population cartograms have also been used as a basis for statistical methods to detect disease clusters. In an early but illustrative example, reported cases of Wilm’s tumor were plotted on a population cartogram and the resulting pattern was compared with that produced by an equal number of randomly placed points [32]. Later researchers applied formal tests of statistical significance to test the hypothesis that event locations on a cartogram of the at-risk population are spatially random [12, 33, 34]. The logic of this approach is that the cartogram translates a null hypothesis of random distribution within the population into a spatially random distribution [35], thus converting a Bernoulli process into an equivalent Poisson process. In this manner, at-risk population cartograms avoid the need for complex statistical methods to handle spatial inhomogeneity [36].

Although the above studies used cartograms as the basis for statistical hypothesis testing, these tests have been automated and are confirmatory in nature. We are not aware of any studies that have employed cartograms within a geovisual exploratory framework to visually communicate uncertainty or statistical significance. This is an important omission, considering that epidemiological maps are probably more useful in exploratory settings than in formal hypothesis testing [1, 37]. With this in mind, we seek methods to visually communicate uncertainty on a cartogram within a geovisual analytics approach that takes advantage of human perception and cognitive abilities to identify patterns and relationships in spatial data [38].

Our use of cartograms to communicate uncertainty is motivated by a natural association between map area and a layperson’s (i.e. informal) concept of significance. From a cartographic perspective, a given event rate is perceived as more indicative of a meaningful pattern if it occurs over a larger map area. In formal hypothesis testing, the statistical significance of an observed rate increases monotonically with and is often (but not always) fully determinated by sample size. Thus, a possible approach to visualizing uncertainty is to designate sample size as the population variable of a cartogram, allowing the map reader to associate the area of a region with its statistical significance.

Given this general framework, the process of exploratory geovisualization begins with the designation of null and alternative hypotheses. In what follows, we assume a null hypothesis of equal probability of occurrence of a health event among all individuals in a population, and a one-tailed alternative hypothesis of higher probability of occurrence in a given region. However, the method can be extended to other hypothesis testing scenarios (e.g. testing for low probability of occurrence, differences in non-ratio values, etc.). Next, it is necessary to obtain data representing the sample size of each district. In what follows, we assume that all cases are reported and therefore the effective sample size is equivalent to the at-risk population.

Once the hypothesis framework and sample population variable have been established, geovisualization is supported by two complementary mathematical procedures that allow dynamic visual inference of the statistical significance of arbitrary regions on a cartogram of the at-risk population. The first procedure computes the minimum size of a region on the cartogram over which a user-designated rate would be statistically significant. The second and converse procedure computes the minimum rate for which a region of a designated size would be statistically significant. Together, these procedures support a visual scanning process and related tools in which the map reader is able to estimate statistical significance of arbitrarily selected regions.

Strictly speaking, both procedures entail the assumption that statistical significance is dependent only on sample size and event rate. We begin with a simple scenario in which this assumption holds, and then proceed to a more realistic scenario in which it does not.

### Scenario one: A priori designated region

We first consider a single region on a map designated a priori as a potential cluster. An example would be a citizen with no prior belief seeks to determine whether or not a disease is prevalent in his or her county of residence. Under the null hypothesis of random distribution, the sampling distribution of the number of disease occurrences within the designated region can be determined from its population. Let *P* and *p* denote the at-risk populations overall and of the designated region, respectively, and let *R* and *r* denote the observed event rates per person overall and in the designated region. Since the map includes the designated region, we compare the designated region with its complement rather than with the entire map to avoid overlap between the regions under comparison. The hypothesis that the actual event rate in the designated region is higher than in the complement to that region can be tested using a standard difference of proportions test, with the following test statistic:

1 $$z = \frac{{\left( {r - R} \right)}}{{\sqrt {R\left( {1 - R} \right)\left( {\frac{1}{p} - \frac{1}{P}} \right)} }}.$$

We solve for *p* to find the minimum population for which a given rate is statistically significant:

2 $$p = \frac{{z^{2} PR\left( {1 - R} \right)}}{{P\left( {r - R} \right)^{2} + z^{2} R\left( {1 - R} \right)}}.$$

Since population density is uniform on the cartogram, the value of *p* can be used to determine the minimum area on the cartogram for which an observed rate *r* would be statistically significant given a predefined significance threshold and corresponding z-score. Alternatively, we can also solve for *r* to determine the minimum rate for which a given population, and thus area on the cartogram, would be statistically significant:

3 $$r = R + z\sqrt {R\left( {1 - R} \right)\left( {1/p - 1/P} \right)} .$$

Although this framework has served as the basis for previous health mapping applications [2, 3], the assumption of an a priori designated region seems incompatible with visual exploratory analysis, which by definition entails searching for undiscovered event clusters that are unknown in advance. For this reason, we also develop an alternative framework based on a scan statistic, which does not assume an a priori designated region.

### Scenario two: A posteriori designated region

Consider a scenario in which an investigator actively searches a map for clusters of events, with no specific region designated a priori. The investigator will naturally seek to identify a region in which the event rate and/or significance is maximal. Since the investigator is able to search all possible regions, an a posteriori test is needed. A standard approach is to define a *scan statistic* as the most extreme (i.e. highest) value of a designated clustering metric observed for a scan window that is moved in a continuous fashion across the map, such that all possible placements of the scan window are examined. Statistical significance is defined as the probability, given a map constructed under the null hypothesis of random spatial distribution, of obtaining a scan statistic as high or higher than the observed scan statistic [39]. Determination of this significance is a difficult problem, and most solutions require computationally intensive Monte Carlo simulation as well as a priori designation of a large but finite set of scan window locations [39, 40]. To support real-time, dynamic geovisual analysis, however, we seek an analytic method for determining the significance of an a posteriori scan statistic without Monte Carlo simulation.

Alm [41] proposes such a method that estimates the significance of a free-moving rectangular scan window within a larger rectangular region. Specifically, Alm’s formulae estimate the probability under a spatially random Poisson process that there will exist a rectangular window of fixed dimensions containing *n* or more events somewhere within a larger rectangular region containing *N* events. Our use of Alm’s formula is made possible due to the fact that the cartogram translates a Bernoulli process into a Poisson process. To simplify, we assume a square study region with area *A* and overall event density *λ*, and a square moving scan window with area *a*. Alm [41] demonstrates that under the assumption of a random Poisson process, the probability *ϕ* that there exists at least one scan window location with *n* or more events can be estimated by the function *f*(*n*), which is defined as follows:

4a $$\mu_{n} = \left( {1 - \frac{\lambda a}{n}} \right)\lambda \sqrt a \left( {\sqrt A - \sqrt a } \right)p_{\lambda a} \left( {n - 1} \right)$$

4b $$\gamma_{n} = \left( {1 - \frac{\lambda a}{n}} \right)\lambda \sqrt a \left( {\sqrt A - \sqrt a } \right)\left( {\mu_{n - 1} - \mu_{n} } \right)e^{{ - \mu_{n} }}$$

4c $$\phi \approx f\left( n \right) = 1 - F_{\lambda a} \left( {n - 1} \right)e^{{ - \left( {\mu_{n} + \gamma_{n} } \right)}}$$

where *F*_*λa*_ denotes the Poisson distribution function with mean *λa*. To support exploratory visual analysis, we seek the minimum scan area *a* for which an observed rate *n*/*a* would be statistically significant, and also the minimum rate *n*/*a* for which a scan window with area *a* would be statistically significant. We are unable to find an analytic solution to these problems. However, the probability *ϕ* logically increases monotonically with both *n* and *n*/*a*. Therefore, an accurate solution can be obtained quickly using a binary search. A slight complication is that while Alm’s functional estimate *f*(*n*) appears valid for all input parameters in which *ϕ* ≪ 1, *f*(*n*) does not always produce valid probability estimates when *ϕ* ≈ 1. Thus, it is necessary to check the validity of input parameters to avoid computational errors. Details on this process are described in the Additional file 1: Appendix.

### Aggregate regions and compactness metric

Since sample size is additive, the above techniques may be applied to aggregate regions formed from more than one district. This is a distinct advantage in exploratory analysis, but in the case of an a posteriori test the freedom to form regions dynamically affects interpretation of significance values. This is because the distribution of a scan statistic is dependent on both the size and shape of the moving scan window. This renders the search space difficult to define, and also the null distribution of the scan statistic difficult to compute. To mitigate this problem, we support qualitative analysis by supplementing reported statistical significance under the assumption of a compact (square) scan window with a measure of compactness to assess the squareness of a user-designated region. We use existing formulas [42] to calculate a shape metric as a function of the moment of inertia *I*_*g*_(*P*) of polygon *P* about its centroid *g*. If one considers a polygon to be composed of infinitely many points, then *I*_*g*_(*P*) is the average squared distance from these points to *g*. In a compact polygon, all locations in *P* will be relatively close to *g*, resulting in a small value of *I*_*g*_(*P*). A compactness index *c*_*mi*_ for a polygon *P* as:

5 $$c_{mi} = \frac{{I_{g} \left( C \right)}}{{I_{g} \left( P \right)}}$$

where *C* denotes a circle equal in area to *P* [42]. The value ranges from 0 (less compact) to 1 (more compact). Since our formulation of statistical significance is based on a square scan window, we modify this slightly by using a square (*S*) instead of a circle in the numerator:

6 $$c_{mi}^{\prime } = \frac{{I_{g} \left( S \right)}}{{I_{g} \left( P \right)}}$$

The ratio of the moments of inertia of a square and circle with equal area is a constant $\frac{\pi }{3}$, so that $c_{mi}^{\prime } = \frac{\pi }{3}c_{mi}$. The theoretical range of $c_{mi}^{\prime }$ is 0–1.047, with a value greater than one indicating a region that is even more compact than the square scan window.

The compactness metric is provided as a check on the validity of the test assumption. The reported significance estimate is considered more reliable if the value of $c_{mi}^{\prime }$ is closer to 1; to the degree that $c_{mi}^{\prime } < 1$, the significance value should be treated with caution. For example, if a “region” is designated as the aggregate of non-adjacent districts located at opposite corners of the study area, the resulting value of $c_{mi}^{\prime }$ would be close to zero. Intuitively, this would indicate that the investigator apparently went to great lengths to form the region, and therefore the statistical test has low validity. In this manner, $c_{mi}^{\prime }$ is used as a surrogate variable to reflect the extensiveness of the search performed by the human investigator.

## Results

A prototype software application was developed to support geovisual exploratory analysis within the above framework. The application was written in C# and Visual Basic (Microsoft Corp.), with mapping and statistical functions from the DotSpatial and MathNet.Numerics open source libraries. The application reads population count and rate data from a standard polygon shapefile. A choropleth map is then constructed automatically using a diverging color scheme in which the central color represents the overall event rate. At present, the application includes three functions for visualization and exploratory analysis. All functions are responsive to user selections of hypothesis-testing framework (a priori vs. a posteriori) and significance threshold (*p* value).

The first function constructs a custom legend to support visual scanning and detection of regions with statistically significant event rates. The legend indicates the minimum area over which an observed rate would be considered statistically significant given the user-selected hypothesis-testing framework and significance threshold.

Second, interactive spatial scan is supported using a square scan window that follows the movement of the mouse. The size (i.e. population) of the scan window is adjustable. As the user scans the map, the event rate in the scan window is computed as the weighted average of the observed rates in districts that intersect the scan window, weighted by the area (i.e. population) of the intersection. Statistical significance is then calculated based on the scan window population and event rate and reported in real time to the user.

Third, exploration of potential clusters is supported by tools for defining custom regions through aggregation. Districts may be aggregated in any manner to form contiguous or non-contiguous regions. Once a region is constructed, the population, average event rate and shape compactness of the region are reported to the user.

**Fig. 1:**
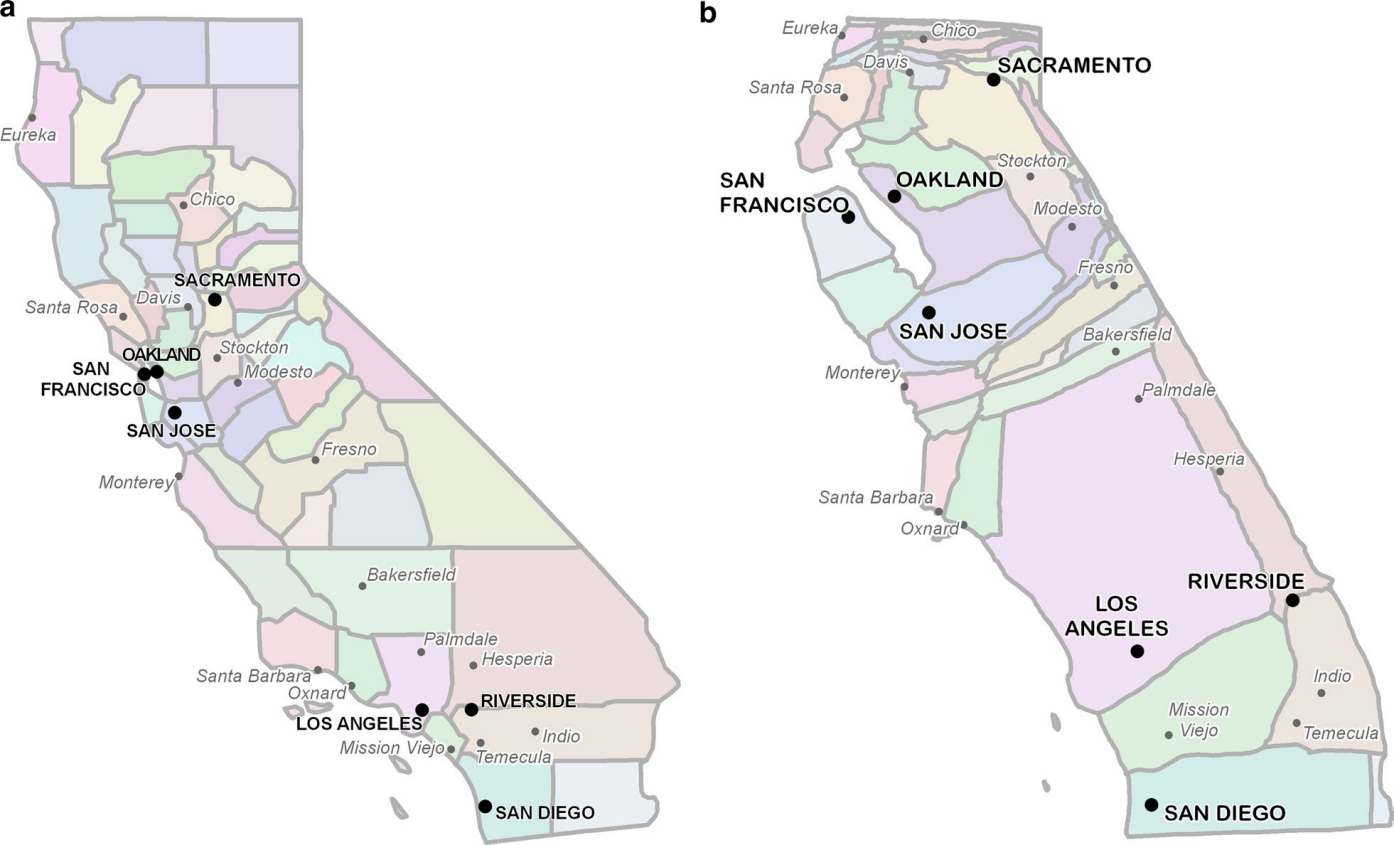
Ordinary map (**a**) and at-risk population cartogram (**b**) of California counties. County sizes on the cartogram are proportional to SEER database age-adjusted leukemia at-risk population values, reported in person-years. Selected cities are shown for reference

To illustrate the geovisual analysis framework, we examine the distribution of age-adjusted leukemia incidence rates for California counties from 2008 to 2013. Data were extracted from the Surveillance, Epidemiology and End Results (SEER 2014-16) program [43]. A cartogram of California was constructed from SEER age-adjusted populations, reported in person-years, using Cartogram Studio software for manual cartogram construction [44]. Figure 1 shows the cartogram alongside a conventional map, with the locations of major cities labelled for reference. Apportionment error is 0.62%, meaning that 99.38% of the population is represented in the correct county on the cartogram.

**Fig. 2:**
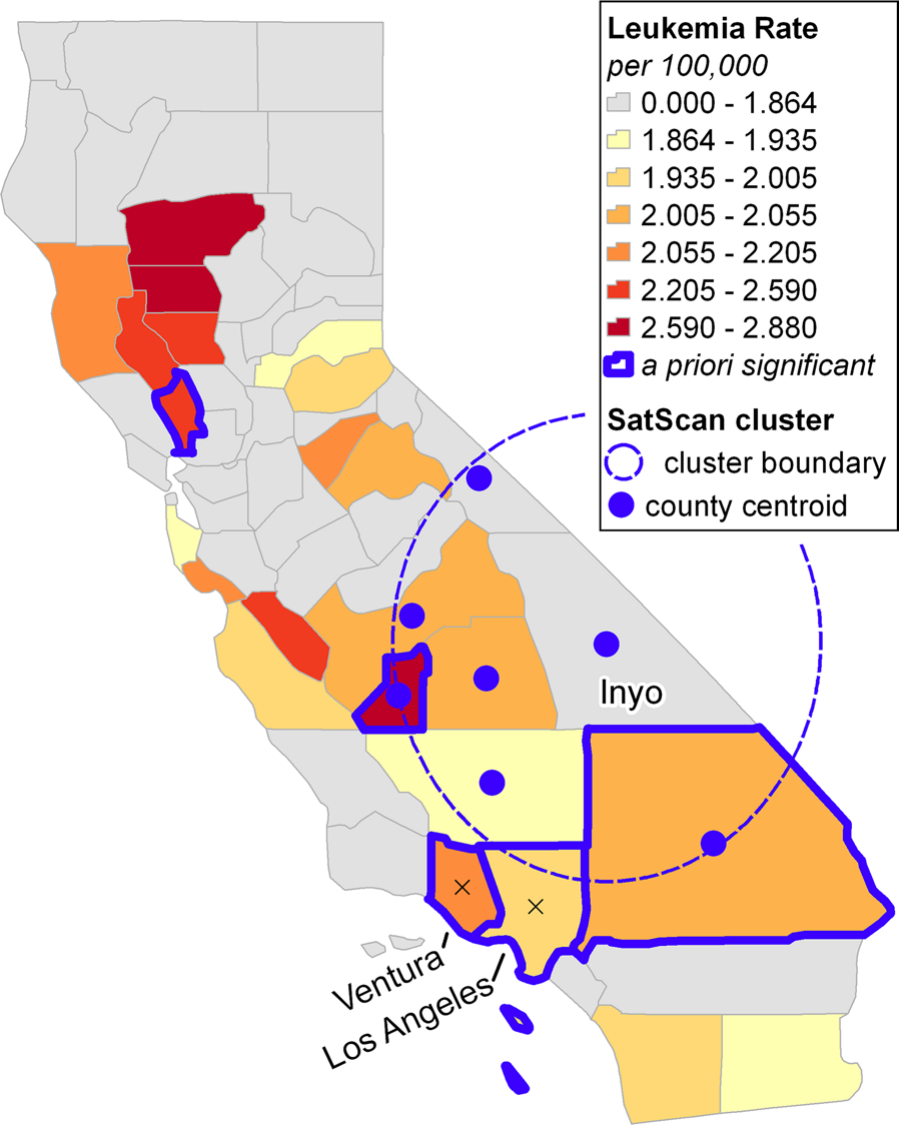
Standard choropleth map of leukemia incidence in California counties, 2008–2013. Counties highlighted in *red outline* have rates that are significantly higher than the remainder of the state according to the a priori test of significance at the 0.05 significance level

Choropleth representation of incidence rates on a conventional map is shown in Fig. 2. Seven classes were constructed by (a) assigning all counties with incidence rates below the overall state rate of 1.864 per 100,000 to a single class, and (b) classifying the remaining counties into six classes using the quantile method. Counties with significantly high rates according to the a priori test are outlined in blue. As a baseline for comparison, results of a Bernoulli spatial scan statistic with circular scan window computed using SaTScan software [45] are also shown in Fig. 2.

There are several limitations to the conventional map. The at-risk population of each county is not shown, so map readers are likely to place cognitive emphasis on counties that are large in area instead of those with high populations. Also, only individual counties are considered as potential clusters, and it is impossible to tell visually whether statistically significant regions could be formed from aggregating counties. Automatic cluster detection using a spatial scan statistic is helpful but idiosyncratic, as it is influenced by the shape and size parameters of the scan window and the spatial arrangement of county centroids. For example, the only statistically significant cluster detected by SaTScan is centered on Inyo County, whose rate is below average. Furthermore, two adjacent counties with rates that are high and statistically significant (Los Angeles, Ventura) are excluded from the cluster (Fig. 2).

**Fig. 3:**
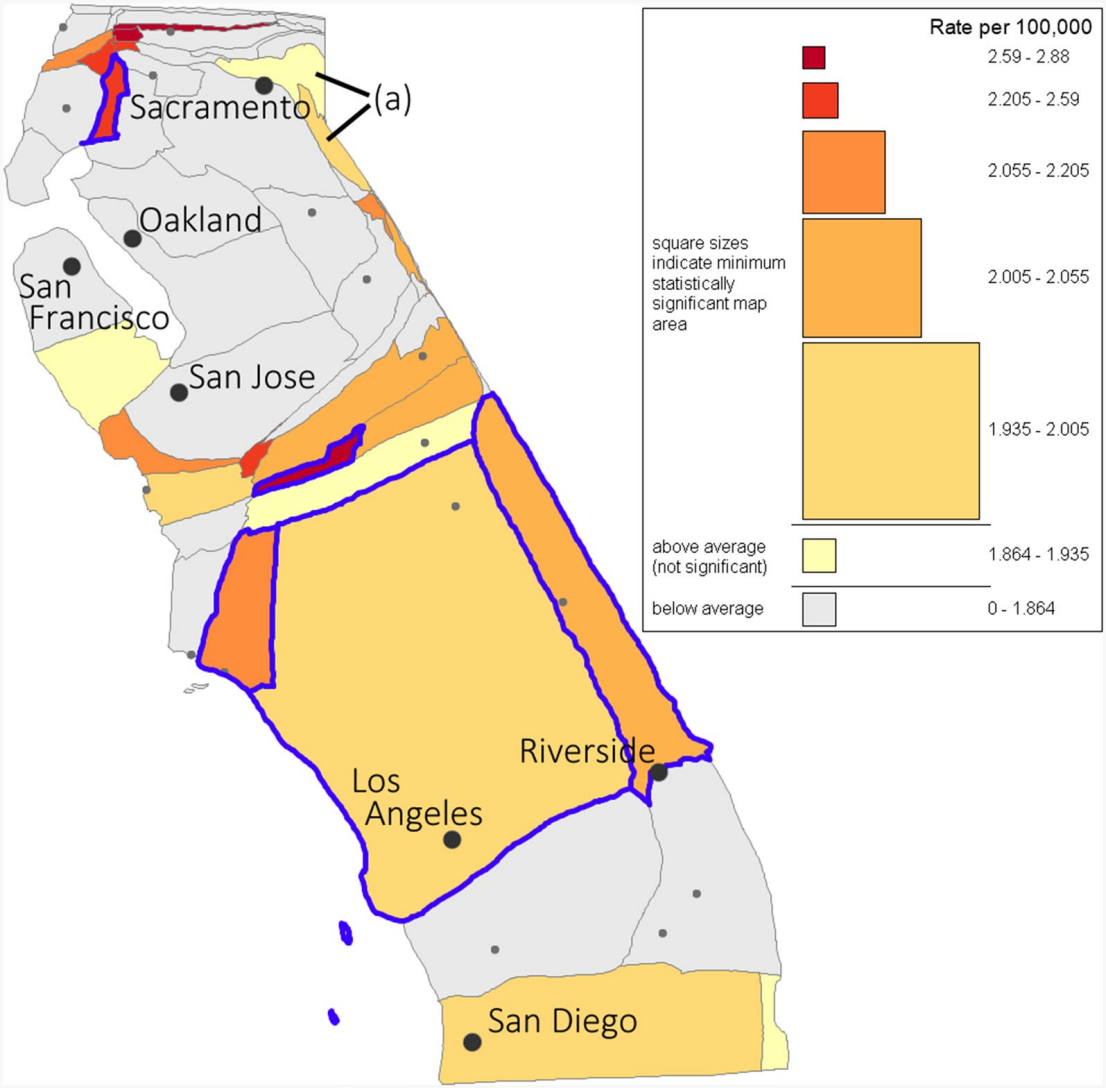
Geovisual analysis environment with a priori test hypothesis and 0.05 significance threshold selected. Legend shows the minimum area for which the observed rate of an a priori designated map region would be significantly higher than the rest of the state

Figure 3 shows the same choropleth map on a cartogram loaded into the geovisual analysis environment with a legend built using the a priori significance test at a threshold of α = 0.05. The legend shows minimum statistically significant areas for counties in each class, which can be compared to the actual counties to infer significance. For example, it can be inferred that the leukemia incidence rate in Los Angeles County, the largest (most populated) county on the map, is moderately higher than the rest of the state but is statistically significant because the county is larger than the size of the corresponding legend symbol of the same color. Statistically significant counties are also highlighted in blue in Fig. 3.

While the geovisual analysis environment allows visual inference of statistical significance for individual districts, the true power of the framework comes from the ability to make inferences about aggregate regions. Note that if the component districts are in different categories, there are multiple corresponding legend symbols and several cases must be considered. If the aggregate region is larger than the symbol associated with the lowest rate category of the component districts, then the aggregate rate is definitely statistically significant. On the other hand, if the aggregate region is smaller than the symbol associated with the highest rate category of the component districts then the aggregate rate is definitely not statistically significant. If neither of the above is true then statistical significance cannot be definitively determined from visual analysis alone. In Fig. 3, the combined area of (a) is clearly smaller than the legend symbol associated with the higher rate (the largest symbol in the legend), and so the aggregate rate of the region formed from these two counties is not statistically significant.

**Fig. 4:**
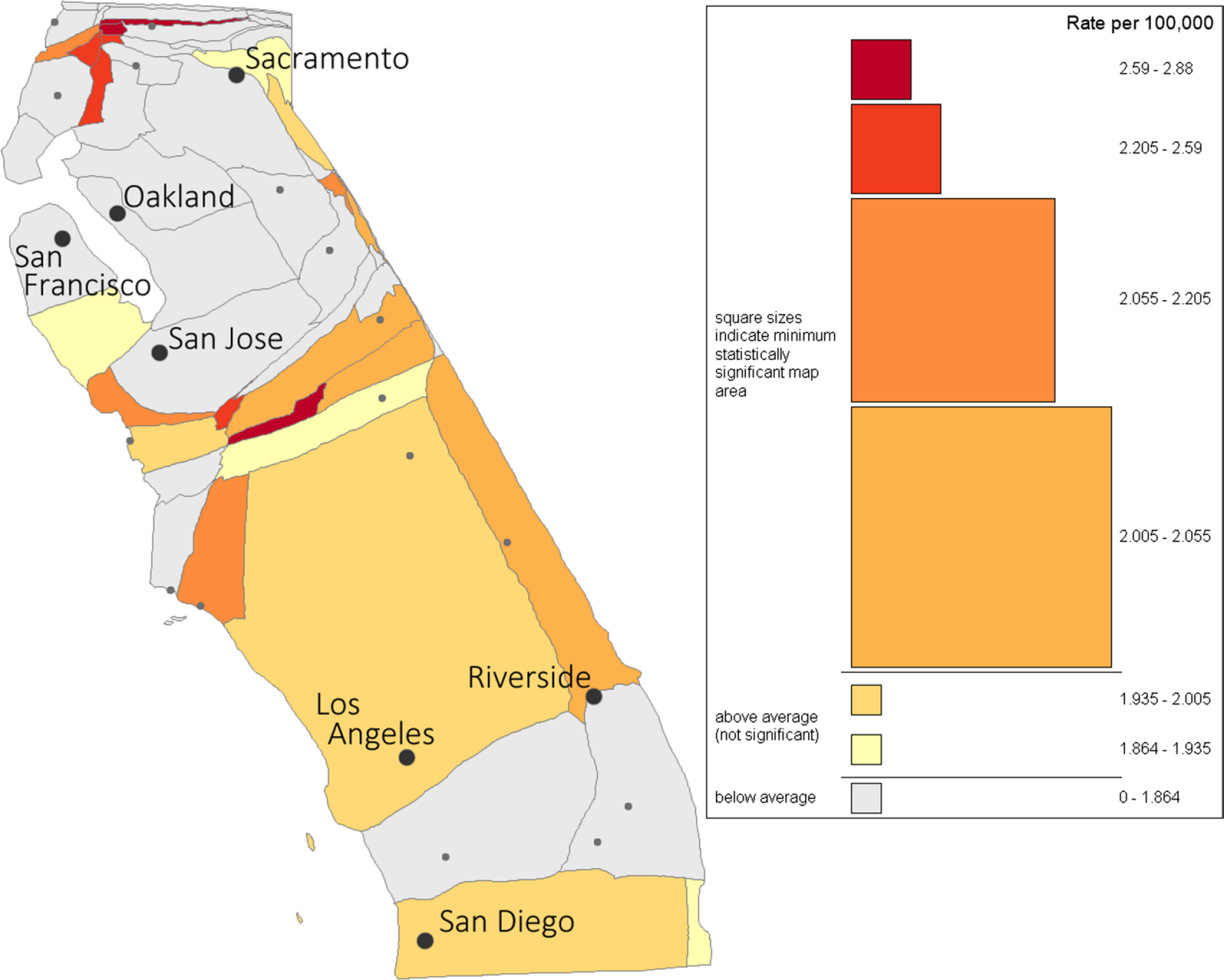
Geovisual analysis environment with global scan hypothesis and 0.05 significance threshold selected. Legend shows the minimum area for which the presence of a square region with the given rate would indicate a statistically significant event cluster

The choice of hypothesis framework has a large influence on statistical significance. Figure 4 shows the same data using the a posteriori test based on a square scan window. It can be seen from the legend in Fig. 4 that the area required for each rate category to be statistically significant increases substantially (as compared to Fig. 3). Visually, it seems clear that none of the counties is individually larger than their corresponding legend symbol. Even aggregating the six northern counties with the highest rates is unlikely to result in a region larger than the average size of the corresponding legend squares. In the southern portion of the state where rates are lower, matching map district colors to the legend suggests that a rather large area would be required to form a statistically significant region.

**Fig. 5:**
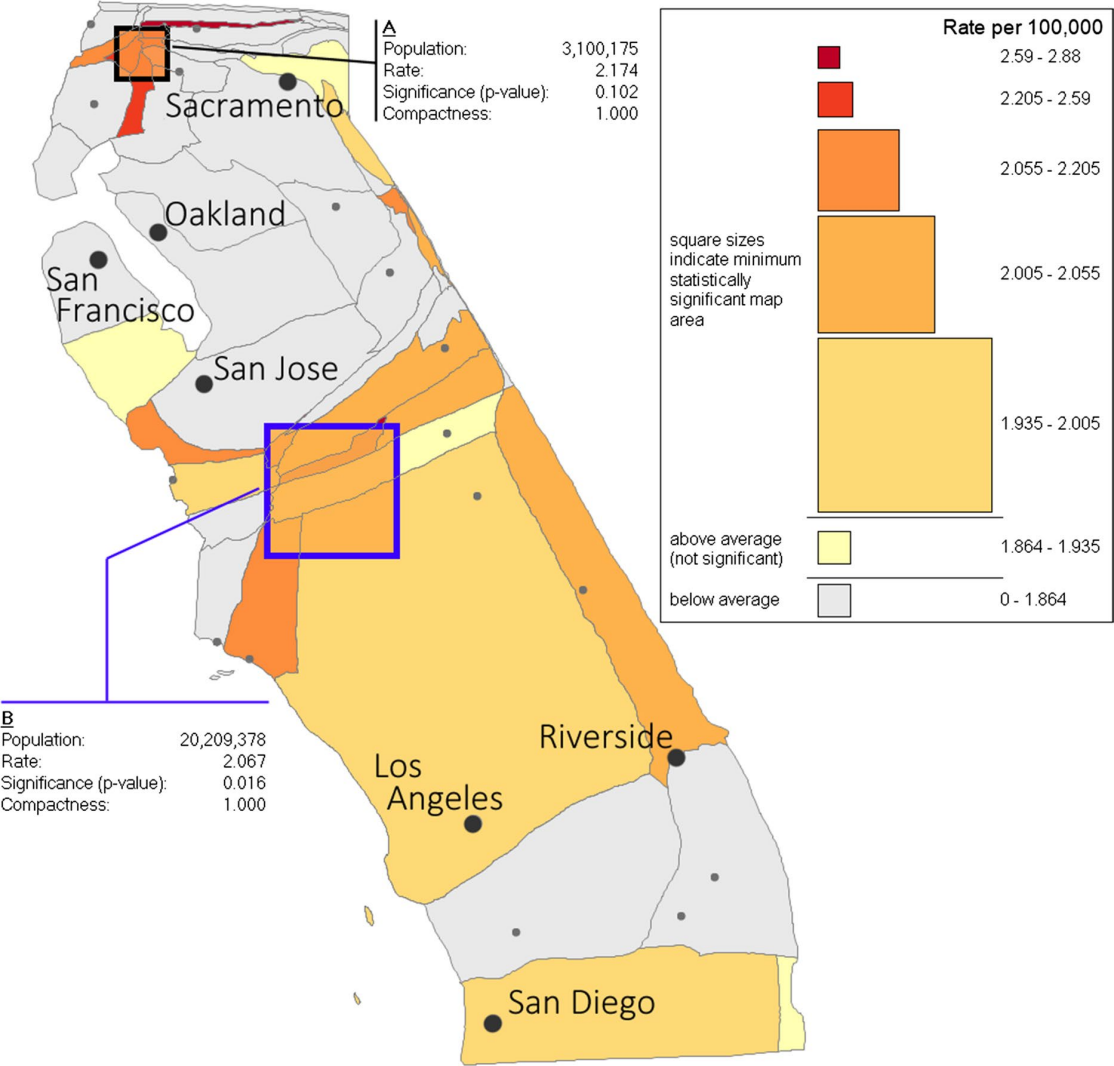
Illustration of two dynamically placed moving scan windows. Reported significance is calculated under the assumption of an a priori designated region. Population sizes of the two regions (A and B) are in person-years

Two examples of the interactive scan process are shown in Fig. 5 under the a priori test. In these examples, scan windows of different sizes have been positioned by the user over potential leukemia cluster locations. The population, combined rate and significance of each scan window are reported dynamically as the mouse is moved, and the color of the scan window is modified to reflect the combined rate. The statistics for scan window A show that the rate in this square region is not statistically higher than the rest of the state (*p* = 0.102). Scan window B covers an area with a lower rate than scan window A, but is larger and therefore statistically significant (*p* = 0.016). To aid in interpretation, the scan window is outlined in blue when the region it covers is statistically significant. It should be noted that the scan windows represent square regions on the cartogram not the map. It would be desirable to transform the scan window into its actual shape in the real world, but this capability requires complete transformation formulae and is not yet implemented in our software.

**Fig. 6:**
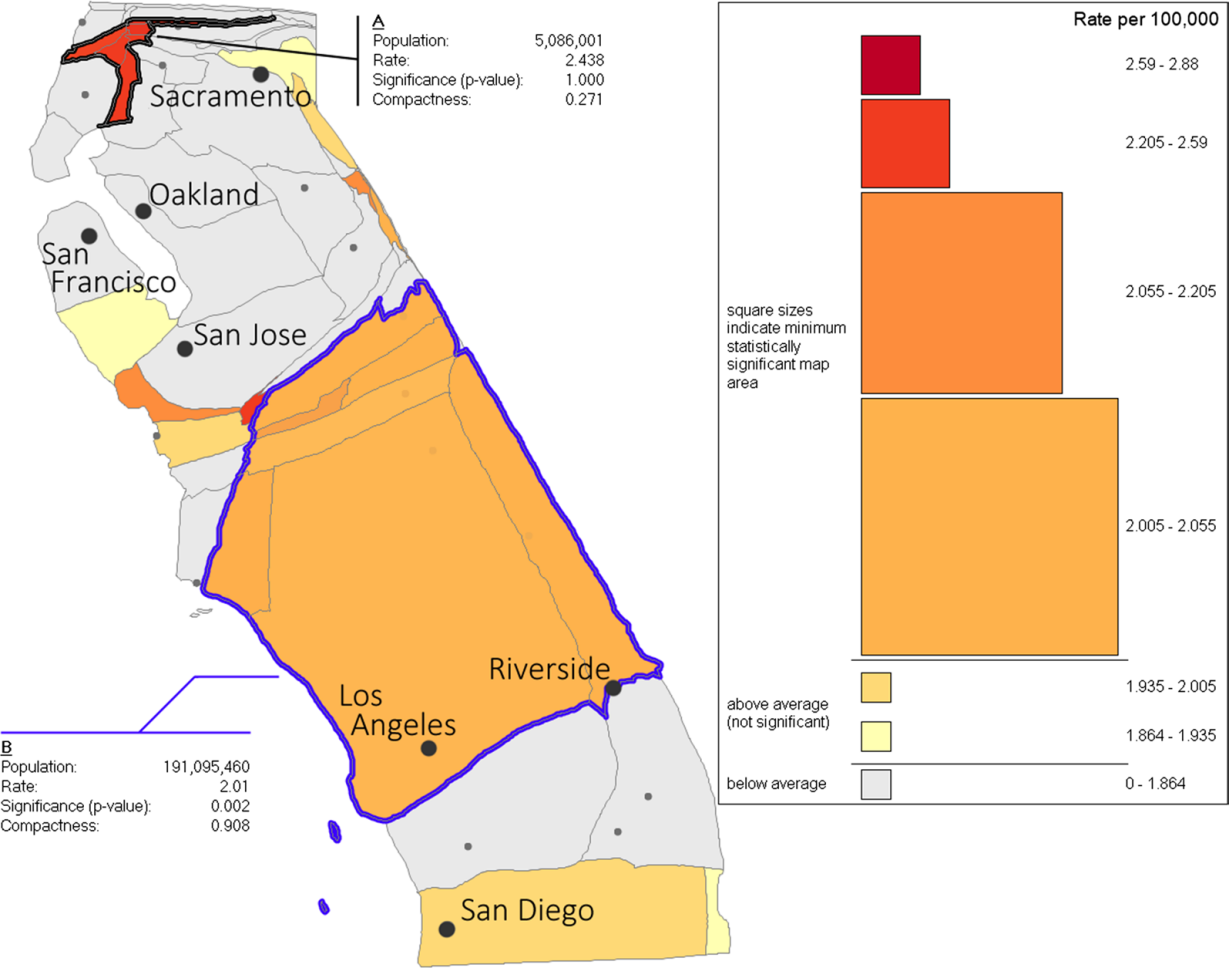
Illustration of two user-defined aggregate regions. Reported significance is calculated using the global scan test under the assumption of a square scan window

Two examples of custom defined aggregate regions are shown in Fig. 6, with significance values calculated using the a posteriori test. Under this stricter test, region A (formed by the six northern counties with above-average leukemia rates) is not statistically significant. Furthermore, the compactness measure for this region is low, suggesting a large departure from the test assumption of a square scan window. However, a statistically significant region can be constructed from several combinations of counties in the southern portion of the state, including the combination comprising region B in Fig. 6. It is notable that all such regions include Los Angeles county despite the fact that its leukemia rate is lower than many nearby counties. This suggests a broad cluster of mildly above average leukemia rates, as opposed to a small cluster of highly elevated rates. Region B in Fig. 6 also has a high compactness index, indicating that its apparent significance is not likely to be due to the relaxation of the assumption of a square scan window.

## Discussion

The cartogram geovisualization approach presented here differs from previous studies on uncertainty visualization in that the latter have focused on methods that apply to individual districts in isolation, either by applying symbol overlays or computing adjusted estimates of disease rates in each district. These approaches do not provide a means to determine uncertainty over larger aggregate regions. If the power of geovisual analytics lies in the elicitation of human cognition and perceptual capabilities, then our approach removes the narrow focus on individual districts and takes full advantage of human cognitive abilities to discern visual patterns in complex data.

The ability to form regions from multiple districts or parts of districts in a highly flexible manner creates a truly exploratory environment that takes full advantage of human powers of pattern recognition. Clusters can be identified that would not be found by spatial scan statistics. Alternatively, automatically detected clusters can be modified by removing districts with low rates or adding districts with high rates that were included/excluded as an artefact of the circular or elliptical scan windows in SaTScan. This is notable because the search space for cluster detection problems is large and grows exponentially with the number of districts if all possible aggregations are considered. If only contiguous regions are considered, the number of possible aggregations is reduced but is still quite large. For example, using the subgraph enumeration algorithm of Wernicke [46], 2,712,603 contiguous regions can be formed from ten or fewer of California’s 58 counties. Thus, relying on algorithmic search may be highly computational intensive and, for larger datasets, practically infeasible. Existing scan techniques handle this problem by restricting the search space based on distance [39] or topological relations [40], but reducing computation time remains a substantial research problem and a barrier to adoption.

Furthermore, restrictions on the search space preclude many potential clusters, including those comprised of non-contiguous or even distant districts, but which share a common property whose relevance might be detected by an expert. For example, a cluster of malnutrition formed from the counties of San Francisco, Los Angeles and San Diego might suggest urban causality, while elevated depression in selected non-contiguous coastal counties might arouse suspicion about the influence of fog. In addition to expert knowledge on potential causal factors, investigators may also carry knowledge of sub-district population patterns that could be used to spot possible clusters that cross district boundaries. For example, in our illustrated examples a health researcher might have further expert knowledge on leukemia rates in different parts of Los Angeles County that is not represented in the data, which might influence the interpretation of the cluster at location B in Figs. 5 and 6. An exploratory framework allows such expert knowledge to be brought to bear. While the geovisual analytics framework presented here has many advantages, it has some drawbacks as well. Although we use significance values to communicate uncertainty on our epidemiological maps (as do many previous researchers), it should be kept in mind that imputed statistical significance values in an exploratory environment cannot be treated as confirmatory in nature. Nevertheless, provision of significance values is an effective way to communicate uncertainty and allows prioritization of regions for further investigation. This is in accordance with the exploratory role of map investigation in epidemiological research, as noted in previous studies.

Another well-known drawback of cartograms is that they are visually unfamiliar and can be aesthetically unappealing. These aesthetics must be weighed against the simple logic of equating map area with statistical significance, which would not be possible without the use of a cartogram. Although we have not yet conducted formal human subjects testing, our experience via presenting these maps to students and colleagues so far suggests that most users are able to quickly learn the fundamental logic of the proposed visualization framework. That is, users are able to visually identify regions and compare them to legend entries to infer statistical significance. However, some details of the inferential processes are less likely to be fully appreciated, including the effects of classification and the appropriate assessment of regions formed from combining districts from different legend classes. By automating the calculation of weighted averages, the interactive tools for scanning and region-building mitigate these issues of interpretation, but further testing and refinement of the visualization environment is needed.

## Conclusion

Although cartograms have been employed previously in health mapping research, the inferential possibilities afforded by equalizing the density of the at-risk population have not been explored in a geovisualization setting. We have demonstrated that cartograms of the at-risk population enable visual determination of statistical significance. Furthermore, since sample size is additive, this determination can be performed for any arbitrary region, assuming a homogenous density within each region. The legend design and interactive scanning and region-building tools presented here work by translating mathematical into visual relationships, allowing inference of statistical significance from the size and observed rate of a region.

## Additional file

**Additional file 1.** Appendix: Checking the Validity of Alm's Functional Estimate.
