# Supplementary material for: Visualizing statistical significance of disease clusters using cartograms
Source: Int J Health Geogr. 2017 May 15;16:19. doi: 10.1186/s12942-017-0093-9 (PMC5433035; doi:10.1186/s12942-017-0093-9)
Supplement: Supplementary file 1 — Additional file 1. Appendix: Checking the Validity of Alm's Functional Estimate. [file 12942_2017_93_MOESM1_ESM.pdf]

Appendix: Checking the validity of Alm's functional estimate

To ensure accuracy, we checked our computations of $f(n)$ (eq. 4) and found them to match exactly the values given in Alm [41]. However, when we plotted values for a wider range of input (fig. A1), it became clear that the estimation function does not yield valid probability estimates everywhere. Logically probability values must fall between 0 and 1, but the calculated value of $f(n)$ was less than zero in several regions. Also, since the objective is to estimate $P(N \geq n)$, probabilities should decrease monotonically with n. From our calculations, however, it became clear that this is the case only for values above the last local maximum (shaded area in fig. A1). The examples provided by Alm [41] all fall within this range. To support a robust interactive software environment where parameters are controlled by user input, however, it is necessary to be able to handle input parameters that fall outside of this range.

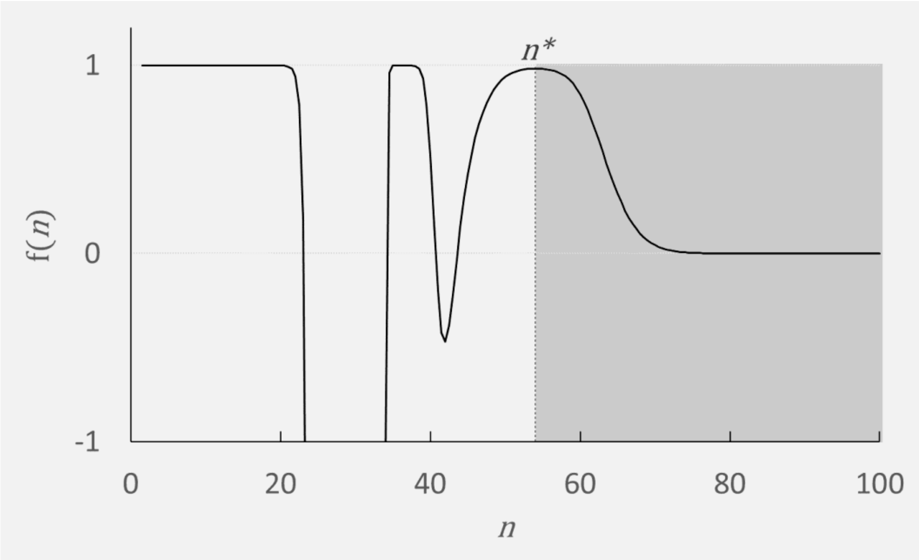

Figure A1: Functional form of $f(n)$. Values shown are for a=100, A=2500, λ=0.4. Shaded area represents valid range of estimates of $P(N \geq n)$.

A simple approach is suggested by the functional form of $f(n)$. Let n^* denote the last value of n for which $f(n)$ is a local maximum. Since $f(n^*) \approx 1$ and $f(n)$ provides accurate estimates for values of $n > n^*$, it seems reasonable to estimate $P(N \geq n)$ as:

$$P(N \geq n) \approx g^*(n) = \begin{matrix} 1 & | & n \leq n^* \\ f(n) & | & n > n^* \end{matrix}$$

Computationally, this requires determination of the value of n^*, which occurs at one of the solutions to $f'(n) = 0$. Unfortunately, the analytic solution to the derivative is intractable.

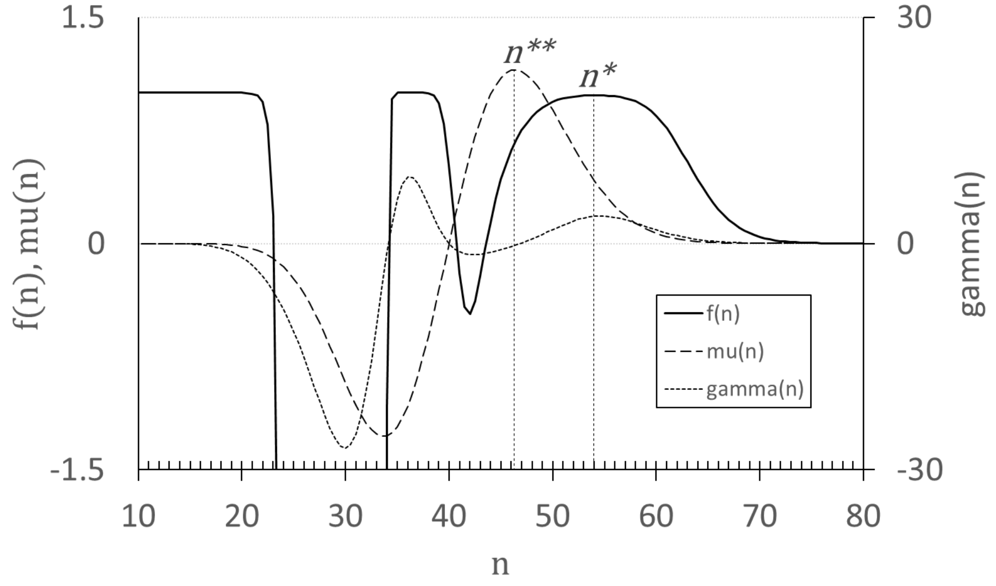

Figure A2: Relationships between $f(n)$, $\mu(n)$ and $\gamma(n)$.

Another approach is discovered by examining the relationships between $f(n)$, $\mu(n)$ and $\gamma(n)$ (fig. A2). Let n^{**} denote the value of n at which the maximum of $\mu(n)$ occurs. We observe that $n^* > n^{**}$, and furthermore $\gamma(n) = 0$ for some value of n between n^{**} and $n^{**} + 1$. From

this, it can be deduced that $f(n)$ increases monotonically between $n^{**}+1$ and n^{*}. If we can determine the value of n^{**}, the probability $P(N \geq n)$ can be estimated as:

$$P(N \geq n) \approx g^{**}(n) = \begin{matrix} f(n) & | & n > n^{**}+1 \text{ and } f'(n) < 0 \\ 1 & | & \text{otherwise} \end{matrix}$$

The value of n^{**} is a solution to:

$$\mu'(n) = 0$$

Determining the derivative and simplifying yields:

$$\psi(n^{**}) - \frac{\lambda a}{(n^{**})^2\left(1 - \frac{\lambda a}{n^{**}}\right)} - ln(\lambda a) = 0$$

where ψ is the Digamma function and ln is the natural logarithm. Substituting the Digamma function with the approximation given by Muqattash and Yahdi [47] using a parameter value of 0.5 yields:

$$ln\left(\frac{n^{**}+0.5}{\lambda a}\right) - \frac{1}{n^{**}-\lambda a} = 0$$

The solution can be determined using Newton's method.

Reference

47. Muqattash I, Yahdi M. Infinite family of approximations of the Digamma function. Mathematical and Computational Modelling. 2006; 43:1329-1336.
